# Supplementary material for: RSV F evolution escapes some monoclonal antibodies but does not strongly erode neutralization by human polyclonal sera
Source: bioRxiv. 2025 Mar 11:2025.03.11.642476. Preprint. [Version 1] doi: 10.1101/2025.03.11.642476 (PMC11952455; doi:10.1101/2025.03.11.642476)
Supplement: Supplement 1 [file NIHPP2025.03.11.642476v1-supplement-1.pdf]

bioRxiv preprint doi: https://doi.org/10.1101/2025.03.11.642476; this version posted March 11, 2025. The copyright holder for this preprint (which was not certified by peer review) is the author/funder, who has granted bioRxiv a license to display the preprint in perpetuity. It is made available under aCC-BY 4.0 International license.

Supplemental Figures

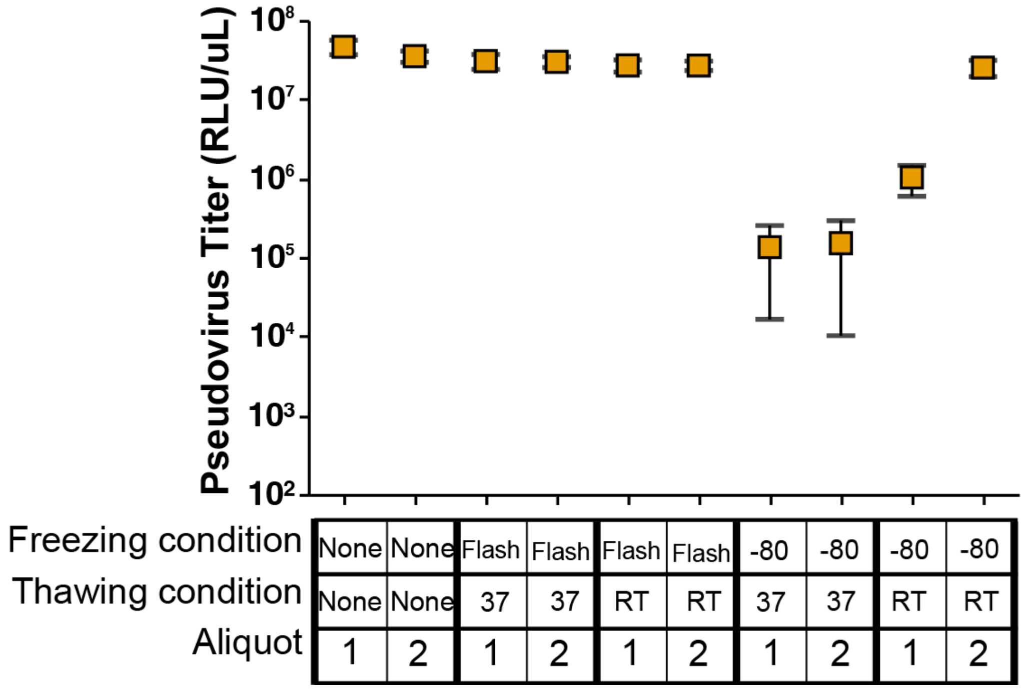

Supplemental Figure 1. Flash freezing on dry ice maintains pseudovirus infectivity.

RSV Long pseudovirus titers (RLU/uL) after different freezing and thawing conditions. Points represent the average titer of two technical replicates. Flash freezing refers to freezing aliquots on dry ice before moving to -80ºC storage. -80 freezing refers to placing aliquots directly in the -80ºC freezer for a slow freeze. Samples in the 37 thawing condition were thawed in a 37ºC water bath while RT samples were thawed at room temperature.

bioRxiv preprint doi: https://doi.org/10.1101/2025.03.11.642476; this version posted March 11, 2025. The copyright holder for this preprint (which was not certified by peer review) is the author/funder, who has granted bioRxiv a license to display the preprint in perpetuity. It is made available under aCC-BY 4.0 International license.

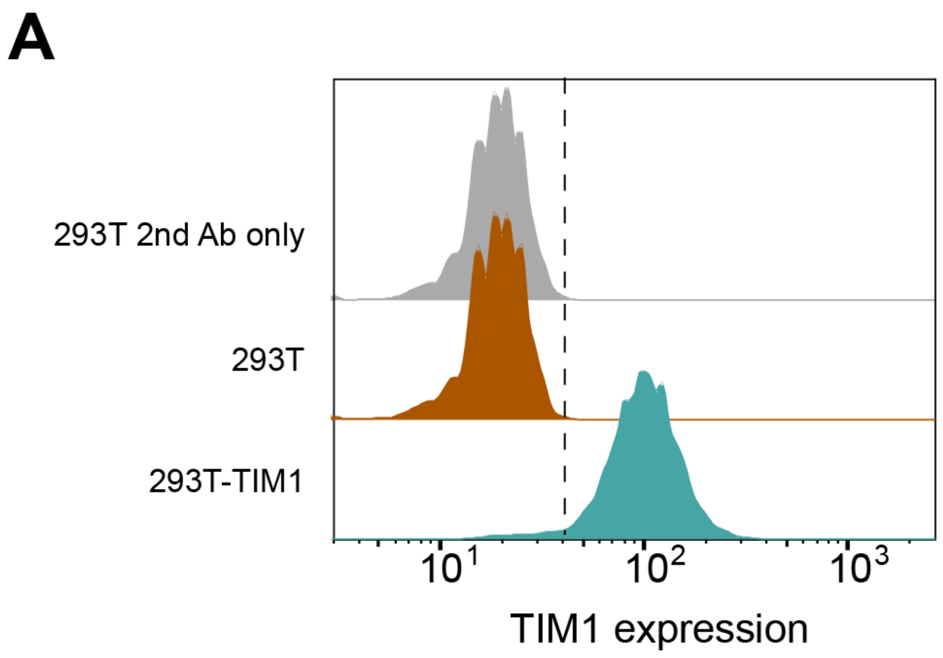

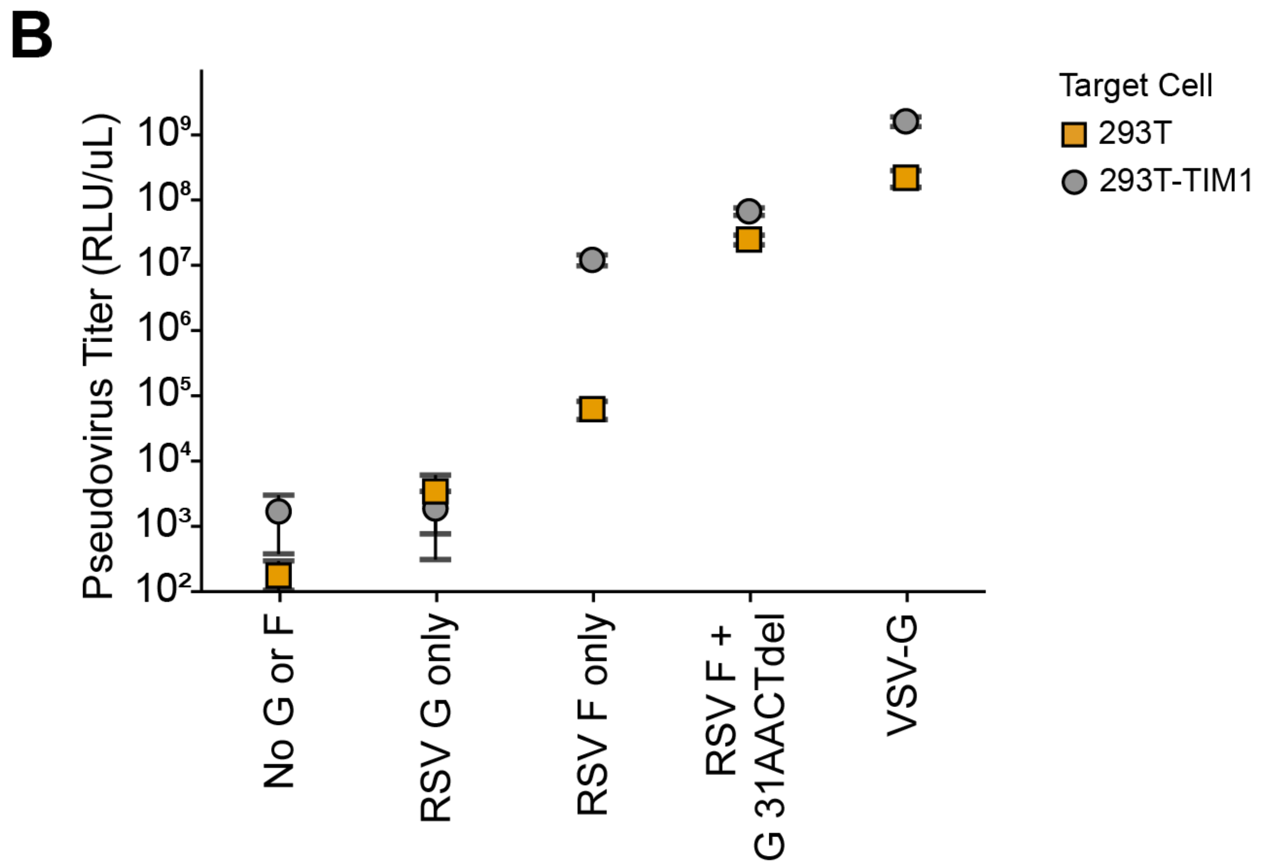

bioRxiv preprint doi: https://doi.org/10.1101/2025.03.11.642476; this version posted March 11, 2025. The copyright holder for this preprint (which was not certified by peer review) is the author/funder, who has granted bioRxiv a license to display the preprint in perpetuity. It is made available under aCC-BY 4.0 International license.

Supplemental Figure 2. RSV G improves pseudovirus titers even on cells expressing TIM1

(A) Immunostaining confirms the expression of TIM1 in 293T-TIM1 cells. Flow cytometry analysis of 293T cells stained with fluorophore-conjugated secondary antibody only (gray), 293T cells stained with anti-TIM1 primary antibody and secondary antibody (orange) and 293T-TIM1 cells stained with anti-TIM1 primary antibody and secondary antibody (teal).

(B) Titers of pseudoviruses expressing the indicated F or G proteins from the Long strain on 293T or 293T-TIM1 target cells. Prior RSV pseudotyping studies have described expression of F alone as being sufficient to produce infectious pseudovirus particles[49–51]. We found that pseudoviruses expressing F alone do infect 293T cells with only low titers, and the addition of the G with a cytoplasmic tail truncation improves titers by 400-fold as shown in Figure 2. This figure shows that infections on 293T-TIM1 cells with pseudovirus expressing F alone results in higher titers, but the addition of the truncated G still improves titers by an additional 6-fold.

bioRxiv preprint doi: https://doi.org/10.1101/2025.03.11.642476; this version posted March 11, 2025. The copyright holder for this preprint (which was not certified by peer review) is the author/funder, who has granted bioRxiv a license to display the preprint in perpetuity. It is made available under aCC-BY 4.0 International license.

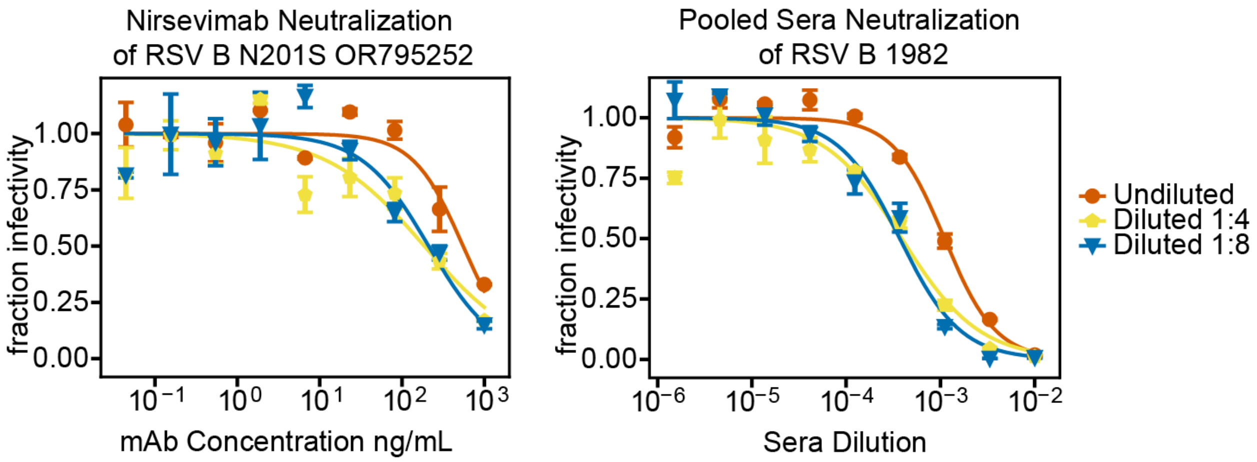

Supplemental Figure 3. Pseudovirus dilution affects neutralization curves

Neutralization curves using undiluted pseudovirus generated from transfection differ from the curves generated using pseudovirus diluted at least 4-fold for the neutralization assay. Neutralization curves for the monoclonal antibody Nirsevimab versus pseudovirus expressing a clinical RSV B F protein (RSV B N201S OR795252) paired with the Long strain G and pooled human sera collected in 2021 versus pseudovirus expressing a clinical RSV B F protein from 1982 paired with the Long strain G. Different colored curves represent pseudovirus supernatant from the transfected producing cells that was undiluted (orange) or diluted in fresh media (1:4 yellow and 1:8 blue) for the neutralization assay. Points indicate the mean ± standard error of two technical replicates. Based on these findings, for all experiments in the paper we used pseudovirus that had been diluted at least 1:4 after its generation by transfection.

bioRxiv preprint doi: https://doi.org/10.1101/2025.03.11.642476; this version posted March 11, 2025. The copyright holder for this preprint (which was not certified by peer review) is the author/funder, who has granted bioRxiv a license to display the preprint in perpetuity. It is made available under aCC-BY 4.0 International license.

A

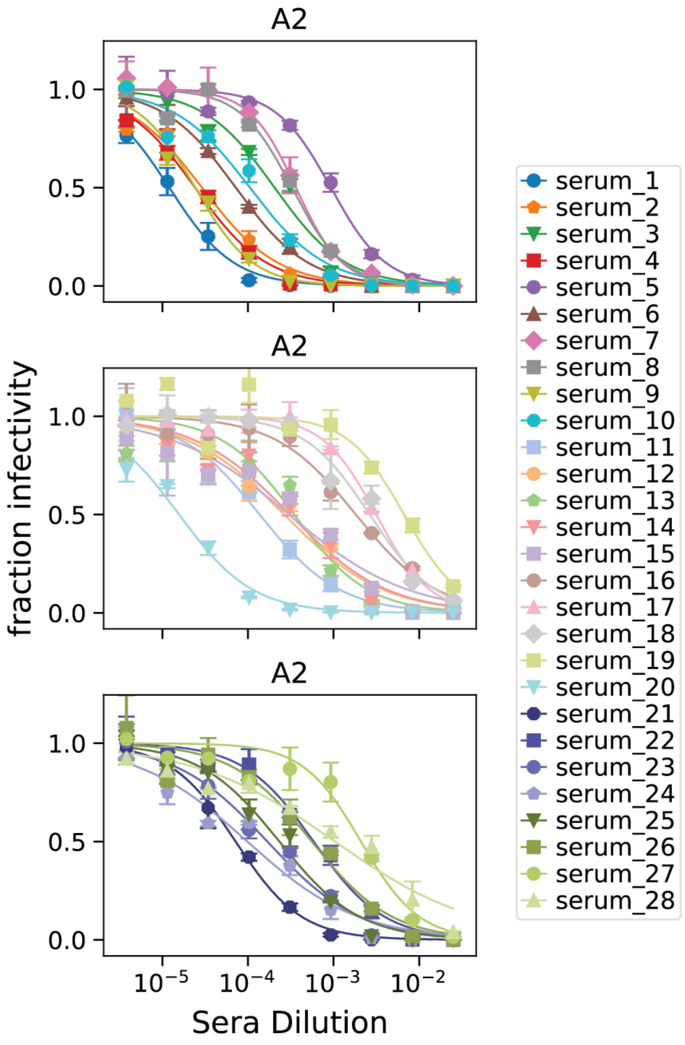

B

Sera	RSV pseudovirus neutralization assay titer	RSV full-length virus neutralization assay titer
1	92188	66231
2	26062	15850
3	4452	7609
4	48302	43386
5	1099	1183
6	12583	5667
7	3693	2195
8	3091	796
9	44700	28349
10	9826	6191
11	7599	3794
12	3500	1982
13	2852	1891
14	2787	1696
15	2808	877
16	453	536
17	376	386
18	413	242
19	154	111
20	62362	29284
21	22193	10727
22	2179	6597
23	4377	4391
24	7686	3622
25	4007	3387
26	1853	1598
27	541	474
28	651	132

Supplemental Figure 4. Comparison of RSV A2 pseudovirus and full-length A2 virus neutralization

(A) Neutralization curves for RSV A2 pseudovirus versus 28 human sera. Points indicate the mean ± standard error of two technical replicates.

(B) Neutralization titers for RSV A2 pseudovirus versus the 28 human sera. RSV pseudovirus neutralization assay titers are the geometric mean of two independent experiments and represent the reciprocal sera dilution at 50% fraction infectivity. RSV A2 full-length virus neutralization assay titers are previously published values and represent the reciprocal sera dilution at 50% fraction infectivity[58]. A correlation plot of these neutralization titers is shown in Figure 3A.

bioRxiv preprint doi: https://doi.org/10.1101/2025.03.11.642476; this version posted March 11, 2025. The copyright holder for this preprint (which was not certified by peer review) is the author/funder, who has granted bioRxiv a license to display the preprint in perpetuity. It is made available under aCC-BY 4.0 International license.

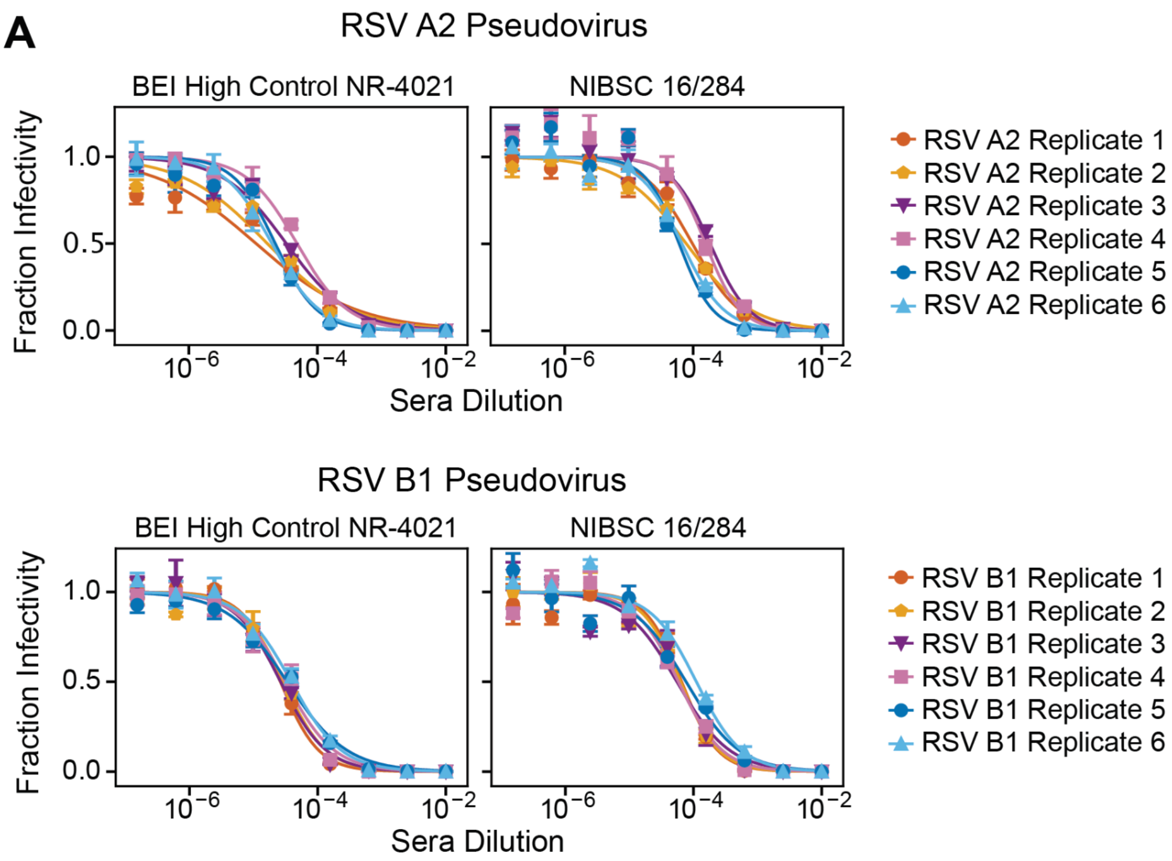

B

Serum	Virus	Number of Replicates	Geometric mean NT50	Conversion Factor for IU/mL
NIBSC 16/284	RSV A2	6	10190	0.196
NIBSC 16/284	RSV B1	6	14444	0.138
				**IU/mL**
BEI High Control NR-4021	RSV A2	6	39187	7691
BEI High Control NR-4021	RSV B1	6	32766	4537
BEI Medium Control NR-4022	RSV A2	2	4092	803
BEI Medium Control NR-4022	RSV B1	2	11950	1655
BEI Low Control NR-4023	RSV A2	2	5851	1148
BEI Low Control NR-4023	RSV B1	2	8432	1167
BEI Negative Control NR-49447	RSV A2	2	>100	
BEI Negative Control NR-49447	RSV B1	2	>100	

Supplemental Figure 5. Neutralization measurements are highly reproducible and can be converted to International Units per milliliter.

(A) Neutralization curves of pseudoviruses with F and G from the RSV A2 or B1 strain by the BEI High reference serum NR4021 and WHO International Reference Antiserum to RSV (NIBSC 16/284) for six independent experimental replicates performed on three separate days by two

bioRxiv preprint doi: https://doi.org/10.1101/2025.03.11.642476; this version posted March 11, 2025. The copyright holder for this preprint (which was not certified by peer review) is the author/funder, who has granted bioRxiv a license to display the preprint in perpetuity. It is made available under aCC-BY 4.0 International license.

different people (Figure 3B shows just two of the replicates for BEI High reference serum NR4021). Points for each replicate indicate the mean ± standard error of two technical repeats of that experiment.

(B) Table detailing neutralization titers for the indicated sera and pseudoviruses. The number of replicates indicates the number of independent experiments for which neutralization titers were measured. The geometric mean NT50 represents the reciprocal sera dilution at 50% fraction infectivity. The measured geometric mean titers for the WHO International Reference Antiserum to RSV (NIBSC 16/284) against RSV A2 and B1 were used to calculate a conversion factor based on the standardized potency of these sera (2000/geometric mean titer). This factor converts measurements from our assay into International Units per milliliter (IU/mL)[59,60]. Namely, to convert titers from our RSV pseudovirus assay into IU/ml, those titers should be multiplied by 0.196 for A2, and 0.183 for B1. Neutralization titers for the BEI Resources reference sera were converted to IU/mL by multiplying the measured geometric mean titer by the corresponding conversion factor. Those titers in IU/ml are then plotted versus the known values for the reference sera in Figure 3C.

bioRxiv preprint doi: https://doi.org/10.1101/2025.03.11.642476; this version posted March 11, 2025. The copyright holder for this preprint (which was not certified by peer review) is the author/funder, who has granted bioRxiv a license to display the preprint in perpetuity. It is made available under aCC-BY 4.0 International license.

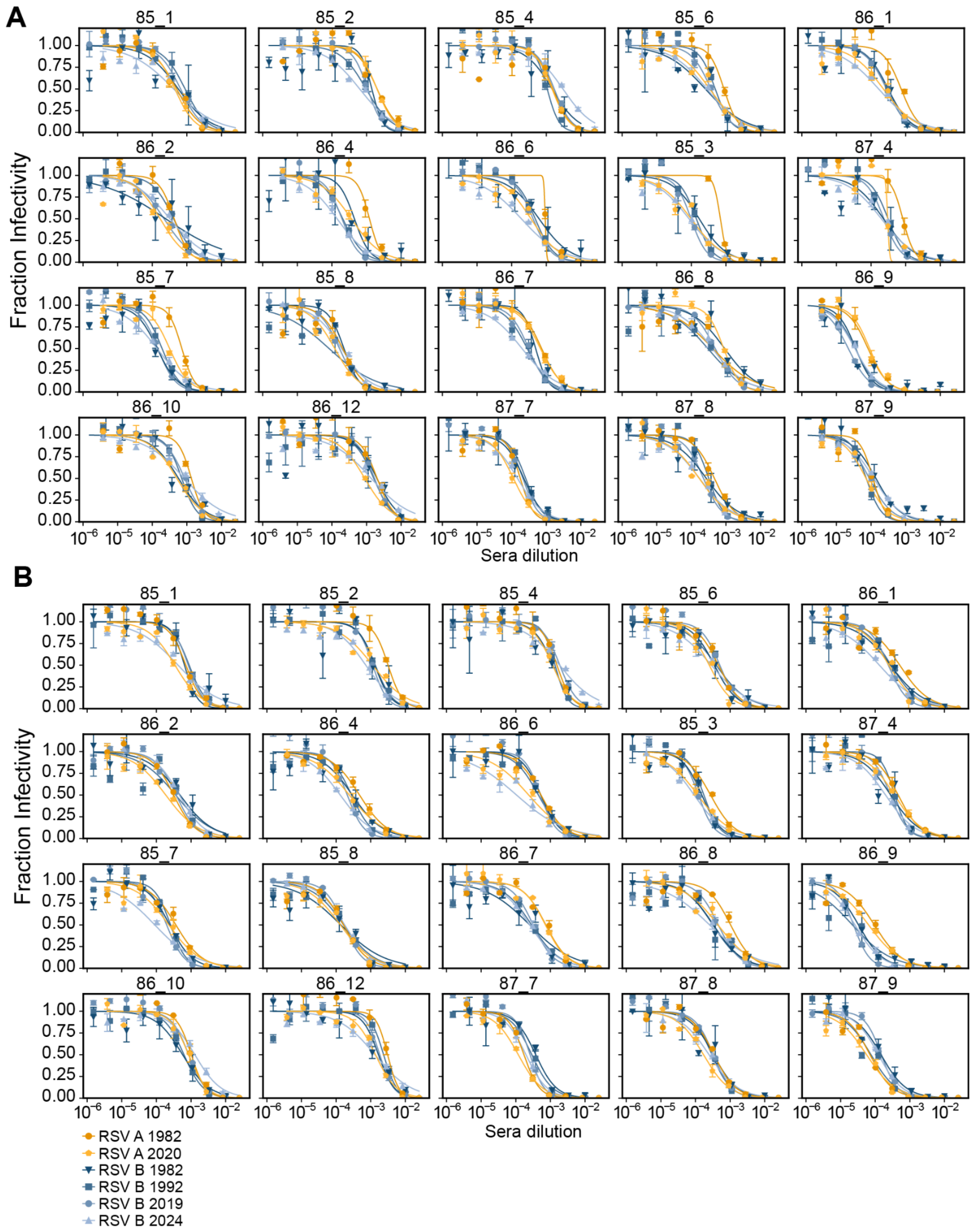

bioRxiv preprint doi: https://doi.org/10.1101/2025.03.11.642476; this version posted March 11, 2025. The copyright holder for this preprint (which was not certified by peer review) is the author/funder, who has granted bioRxiv a license to display the preprint in perpetuity. It is made available under aCC-BY 4.0 International license.

Supplemental Figure 6. Neutralization curves for historical sera versus RSV pseudoviruses

Neutralization curves for historical serum specimens collected from healthy adults in 1985-1987 (depleted for G-binding antibodies) versus RSV pseudoviruses with G from the Long strain and F from either an historical (eg, 1982) and a recent (eg, 2020-2024) subtype A or B strains. Points indicate the mean ± standard error of technical repeats of experiments performed on the same day. Independent experimental replicates performed on different days are indicated by A and B. A summary plot of the neutralization titers is shown in figure 6B.

bioRxiv preprint doi: https://doi.org/10.1101/2025.03.11.642476; this version posted March 11, 2025. The copyright holder for this preprint (which was not certified by peer review) is the author/funder, who has granted bioRxiv a license to display the preprint in perpetuity. It is made available under aCC-BY 4.0 International license.

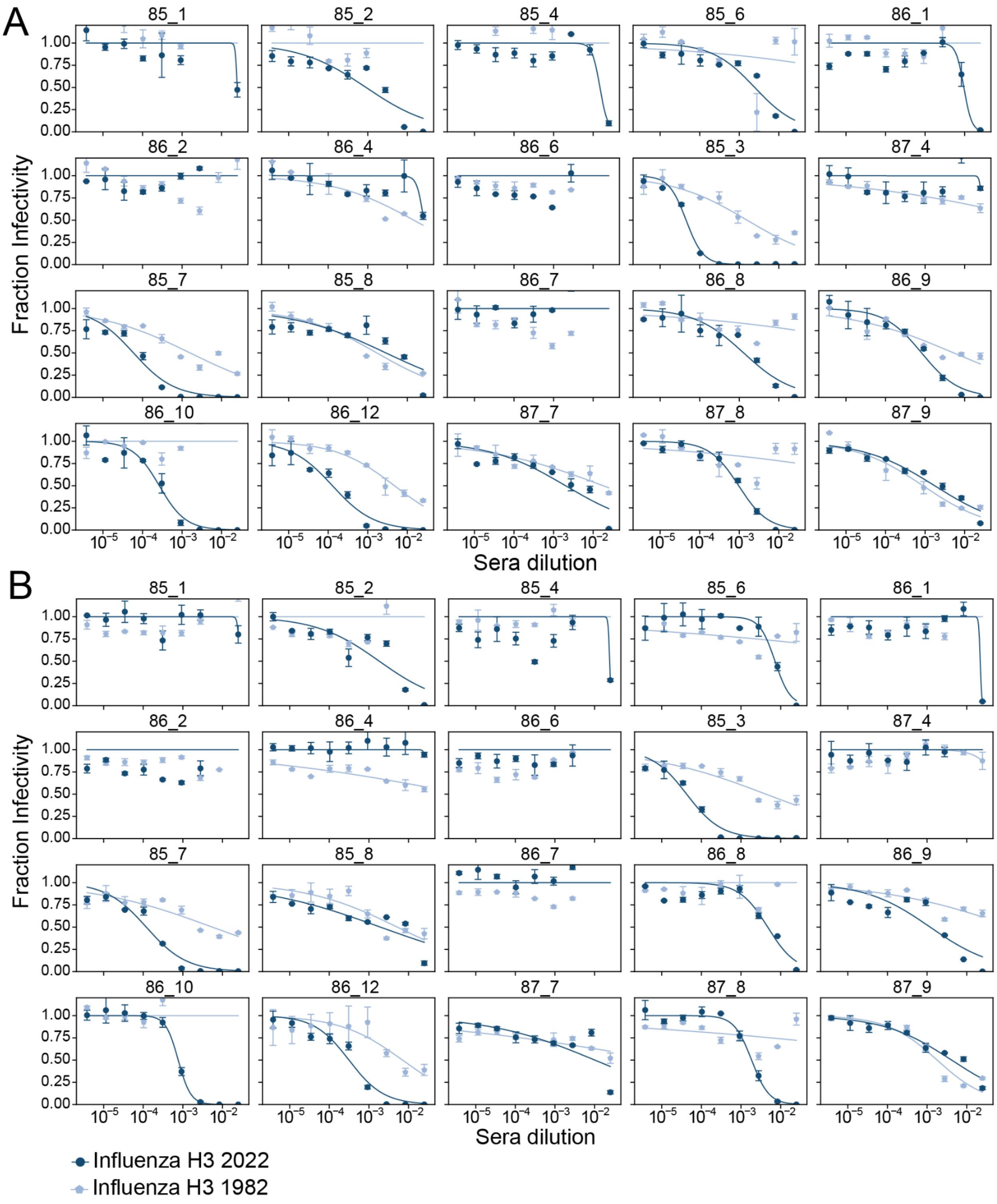

bioRxiv preprint doi: https://doi.org/10.1101/2025.03.11.642476; this version posted March 11, 2025. The copyright holder for this preprint (which was not certified by peer review) is the author/funder, who has granted bioRxiv a license to display the preprint in perpetuity. It is made available under aCC-BY 4.0 International license.

Supplemental Figure 7. Neutralization curves for historical sera versus pseudoviruses expressing influenza hemagglutinin

Neutralization curves for historical serum specimens collected from healthy adults in 1985-1987 versus pseudovirus expressing hemagglutinin (A/Netherlands/233/1982) and neuraminidase (A/HongKong/1/1968) from historical strains versus pseudovirus expressing hemagglutinin and neuraminidase from a recent (A/Massachusetts/18/2022) strain of human H3N2 influenza. Points indicate the mean ± standard error of technical repeats of experiments performed on the same day. Independent experimental replicates performed on different days are indicated by A and B. A summary plot of the neutralization titers is shown in Figure 6C.

bioRxiv preprint doi: https://doi.org/10.1101/2025.03.11.642476; this version posted March 11, 2025. The copyright holder for this preprint (which was not certified by peer review) is the author/funder, who has granted bioRxiv a license to display the preprint in perpetuity. It is made available under aCC-BY 4.0 International license.

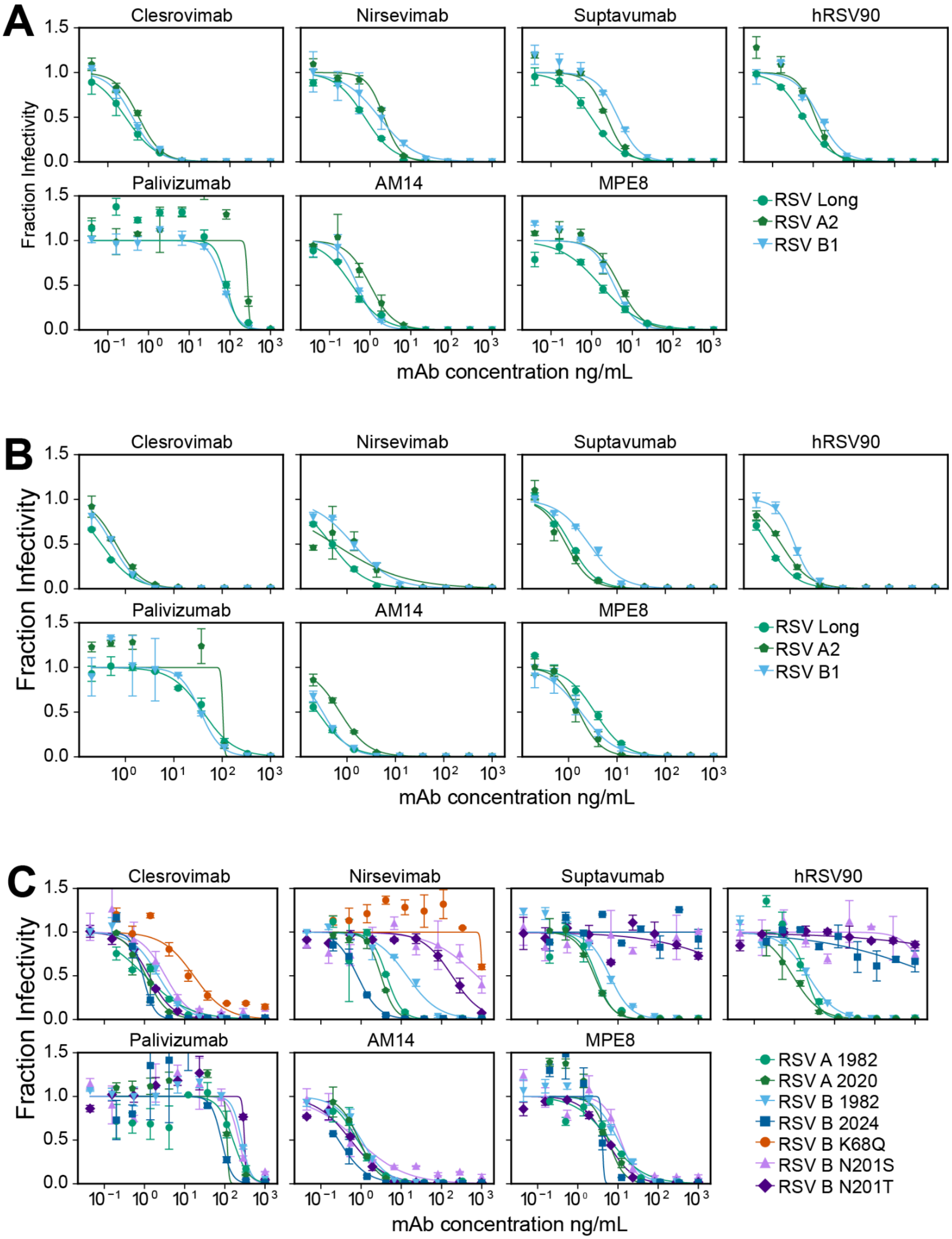

bioRxiv preprint doi: https://doi.org/10.1101/2025.03.11.642476; this version posted March 11, 2025. The copyright holder for this preprint (which was not certified by peer review) is the author/funder, who has granted bioRxiv a license to display the preprint in perpetuity. It is made available under aCC-BY 4.0 International license.

Supplemental Figure 8. Neutralization curves for monoclonal antibodies versus RSV pseudoviruses expressing F and G from lab-adapted strains

(A and B) Neutralization curves for monoclonal antibodies versus RSV pseudoviruses F and G from lab-adapted strains Long, A2 and B1. Points indicate the mean ± standard error of technical repeats of experiments performed on the same day. A and B represent independent experimental replicates performed on different days.

(C) Neutralization curves for monoclonal antibodies versus pseudoviruses expressing the indicated F protein paired with Long G. Points indicate the mean ± standard error of technical repeats of experiments performed on the same day. Neutralization curves for these monoclonal antibodies from a separate experiment performed on a different day are in Figure 7.

bioRxiv preprint doi: https://doi.org/10.1101/2025.03.11.642476; this version posted March 11, 2025. The copyright holder for this preprint (which was not certified by peer review) is the author/funder, who has granted bioRxiv a license to display the preprint in perpetuity. It is made available under aCC-BY 4.0 International license.

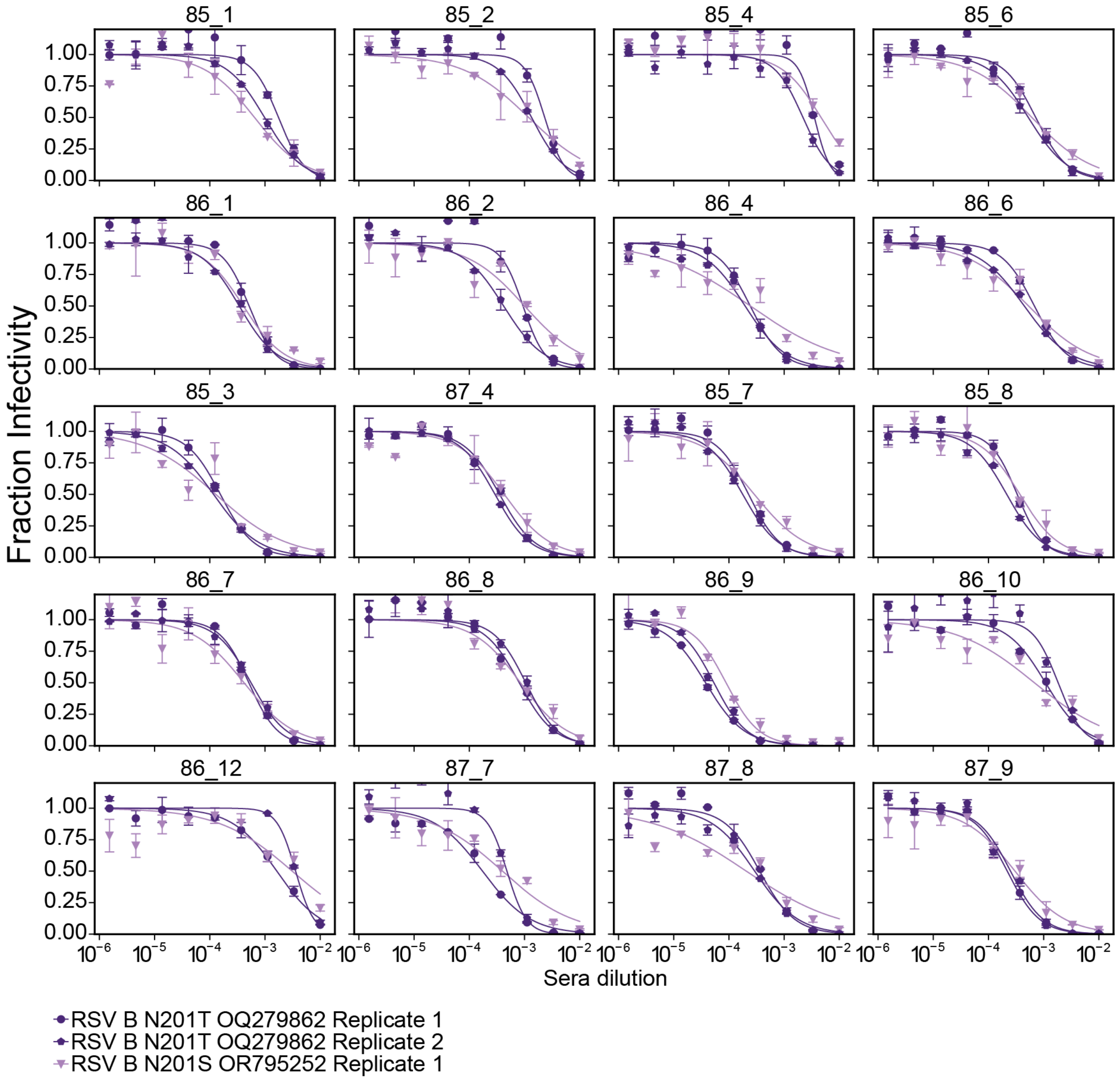

Supplemental Figure 9 . Neutralization curves for historical sera versus RSV pseudoviruses expressing F with nirsevimab resistance mutations

Neutralization curves for historical serum specimens collected from healthy adults in 1985-1987 (depleted for G-binding antibodies) versus RSV pseudoviruses with G from the Long strain and F with known nirsevimab escape mutations. Points indicate the mean ± standard error of technical repeats of experiments performed on the same day. Two independent experimental replicates performed on different days are shown for RSV B N201T OQ279862. One experimental replicate was performed and is shown for RSV B N201S OR795252. A summary plot of the neutralization titers is shown in Figure 7C.

bioRxiv preprint doi: https://doi.org/10.1101/2025.03.11.642476; this version posted March 11, 2025. The copyright holder for this preprint (which was not certified by peer review) is the author/funder, who has granted bioRxiv a license to display the preprint in perpetuity. It is made available under aCC-BY 4.0 International license.

bioRxiv preprint doi: https://doi.org/10.1101/2025.03.11.642476; this version posted March 11, 2025. The copyright holder for this preprint (which was not certified by peer review) is the author/funder, who has granted bioRxiv a license to display the preprint in perpetuity. It is made available under aCC-BY 4.0 International license.

Supplemental Tables

Supplemental Table 1. Sequence accession numbers and pseudovirus names. Plasmid sequences can be found at https://github.com/jbloomlab/RSV-evolution-neut/tree/main/04_plasmid_maps.

Pseudovirus name	Viral protein	GenBank Accession/GISAID ID	Comments
RSV Long	RSV Long F	AY911262.1	
	RSV Long G	AY911262.1	
RSV A2	RSV A2 F	QGW56794.1	
	RSV A2 G	KT992094.1	
RSV B1	RSV B1 F	AF013254	also referred to as RSV BWV40167-85
	RSV B1 G	AF013254	also referred to as RSV BWV40167-85
RSV A 1982	RSV A 1982 F	KJ723472	paired with RSV Long G 31AA CTdel to make RSV pseudovirus
RSV A 2020	RSV A 2020 F	PP495954	
RSV B 1982	RSV B 1982 F	MG642043	
RSV B 1992	RSV B 1992 F	OK649748	
RSV B 2019	RSV B 2019 F	PP352433	
RSV B 2024	RSV B 2024 F	PP660445	
RSV B K68Q MN365362	RSV B K68Q MN365362 F	MN365362	
RSV B N201S OR795252	RSV B N201S OR795252 F	OR795252	
RSV B N201T OQ279862	RSV B N201T OQ279862 F	OQ279862	
Influenza H3 2022	A/Massachusetts/18/2022 (H3N2) HA	EPI2413620	GISAID ID
	A/Massachusetts/18/2022 (H3N2) NA	EPI2413618	GISAID ID

bioRxiv preprint doi: https://doi.org/10.1101/2025.03.11.642476; this version posted March 11, 2025. The copyright holder for this preprint (which was not certified by peer review) is the author/funder, who has granted bioRxiv a license to display the preprint in perpetuity. It is made available under aCC-BY 4.0 International license.

Influenza H3 1982	A/Netherlands/233/1982 (H3N2) HA	EPI545279	GISAID ID
	A/HongKong/1/1968 (H3N2) NA	AF348184.1	GenBank ID
VSV-G	VSV-G		addgene plasmid #12259

bioRxiv preprint doi: https://doi.org/10.1101/2025.03.11.642476; this version posted March 11, 2025. The copyright holder for this preprint (which was not certified by peer review) is the author/funder, who has granted bioRxiv a license to display the preprint in perpetuity. It is made available under aCC-BY 4.0 International license.

Supplemental Table 2. Sera identifiers, collection year and age at collection for the 20 historical sera specimens.

sera ID	collection year	patient age
85_1	1985	27
85_2	1985	20
85_3	1985	26
85_4	1985	23
85_6	1985	40
85_7	1985	unknown
85_8	1985	unknown
86_1	1986	50
86_10	1986	unknown
86_12	1986	unknown
86_2	1986	23
86_4	1986	25
86_6	1986	32
86_7	1986	unknown
86_8	1986	unknown
86_9	1986	unknown
87_4	1987	22
87_7	1987	unknown
87_8	1987	unknown
87_9	1987	unknown
